# Unveiling the Potential of Vitamin D3 Orodispersible Films: A Comprehensive FTIR and UV–Vis Spectroscopic Study

**DOI:** 10.3390/molecules29163762

**Published:** 2024-08-08

**Authors:** Alfio Torrisi, Mariapompea Cutroneo, Lorenzo Torrisi, Salvatore Lavalle, Alessia Forzina, Francesco Pegreffi

**Affiliations:** 1 Department of Medicine and Surgery, Kore University of Enna, 94100 Enna, Italy; salvatore.lavalle@unikore.it (S.L.); francesco.pegreffi@unikore.it (F.P.); 2 Department of Mathematics and Computer Sciences, Physical Sciences and Earth Sciences (MIFT), Messina University, 98166 Messina, Italy; mariapompea.cutroneo@unime.it (M.C.); lorenzo.torrisi@unime.it (L.T.); 3 IBSA Institut Biochimique SA, CH-6912 Lugano, Switzerland; alessia.forzina@ibsa.ch

**Keywords:** vitamin D3, orodispersible films, ATR-FTIR spectroscopy, UV–Vis–NIR spectroscopy, UV irradiation

## Abstract

Vitamin D3 is a crucial fat-soluble pro-hormone essential for bolstering bone health and fortifying immune responses within the human body. Orodispersible films (ODFs) serve as a noteworthy formulation strategically designed to enhance the rapid dissolution of vitamin D, thereby facilitating efficient absorption in patients. This innovative approach not only streamlines the assimilation process but also plays a pivotal role in optimizing patient compliance and therapeutic outcomes. The judicious utilization of such advancements underscores a paradigm shift in clinical strategies aimed at harnessing the full potential of vitamin D for improved patient well-being. This study aims to examine the vitamin D3 ODF structure using spectroscopic techniques to analyze interactions with excipients like mannitol. Fourier-transform infrared spectroscopy (FTIR) and ultraviolet–visible (UV–Vis) spectroscopy were utilized to assess molecular composition, intermolecular bonding, and vitamin D3 stability. Understanding these interactions is essential for optimizing ODF formulation, ensuring stability, enhancing bioavailability, and facilitating efficient production. Furthermore, this study involves a translational approach to interpreting chemical properties to develop an administration protocol for ODFs, aiming to maximize absorption and minimize waste. In conclusion, understanding the characterized chemical properties is pivotal for translating them into effective self-administration modalities for Vitamin D films.

## 1. Introduction

Among the various excipients utilized in orodispersible film (ODF) formulations, vitamin D3 stands out for its crucial role in enhancing bioavailability. Vitamin D represents a group of five different fat-soluble vitamins: D1, D2, D3, D4, and D5. The human body can recognize only two of these five vitamins: vitamin D2 (ergocalciferol) and vitamin D3 (cholecalciferol). The primary and widely recognized function of vitamin D3 is to facilitate calcium absorption, thereby ensuring bone integrity and mitigating bone fragility. Beyond its skeletal functions, vitamin D3 acts as a hormone regulating various physiological processes, including immune response modulation and inflammation regulation [1]. Deficiency in vitamin D3 is linked to numerous health issues, such as diabetes, cardiovascular diseases, Alzheimer’s disease, and asthma [2,3]. Being a fat-soluble vitamin, vitamin D3 is mostly found in fatty fish (such as salmon, mackerel, and cod liver oil), egg yolk, and liver. Even sun exposure is able to promote the production of this vitamin, which is why D3 is known as the “sun vitamin”. In regions with limited sunlight, it is recommended to take supplements containing vitamin D3, especially in the winter months. ODFs have garnered substantial attention in pharmaceutical investigation owing to their distinctive characteristics, notably their rapid dissolution in the oral cavity. This feature is particularly convenient for active individuals and populations with swallowing difficulties, such as the elderly, children, or those unable to swallow tablets, given that no water is required for their assimilation. In this work, we studied the structure of vitamin D3 orodispersible films utilizing Fourier Transform Infrared Spectroscopy (FTIR) and Ultraviolet-Visible (UV–Vis) spectroscopic techniques. FTIR spectroscopy provides valuable insights into the molecular composition and interactions within the film matrix, elucidating the vibrational modes of chemical bonds present. On the other hand, UV–VIS spectroscopy offers a powerful tool for assessing the electronic transitions and optical properties of active pharmaceutical ingredients such as vitamin D3. This study investigates potential hydrogen bonding, molecular rearrangements, and crystalline transformations induced during the film formulation and dissolution of vitamin D3 ODFs.

The UV–Vis spectroscopy will be employed to assess the spectral characteristics of vitamin D3 in the presence of mannitol and other excipients, shedding light on any potential interactions or degradation pathways under simulated environmental conditions. Literature reports on the physical and chemical characterization of such ODF vitamins, maintaining high stability at different environmental conditions, which remains higher than 90% even after 3 months at 40 °C [3]. It has already been demonstrated that the vitamin D3 stability in aqueous media depends on several parameters such as temperature, light, oxygen, pH, concentration, and metal ions [4]. Some of these aspects will be investigated in this work.

## 2. Results and Discussion

**Figure 1:**
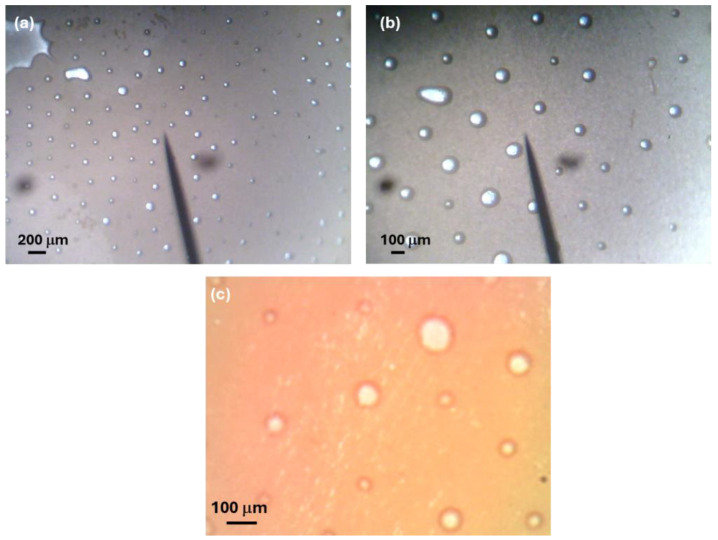
Optical microscope photos of the vitamin D3 orodispersible film (ODF) at (**a**) 10×, (**b**) 20× (in gray contrast color), and (**c**) at 50× (real sample color), showing the irregular circular porosity of the surface.

We characterized 2000 UI vitamin D3 films from IBSA^®^ [5], based on FilmTec^®^ technology [6] (Lugano, Switzerland), having a rectangular area of 2 × 2.9 cm^2^ and a thickness of about 118 microns. Given the area of 5.8 cm^2^, an average thickness of 118 microns of the sample, and its weight of 0.1 g, the density of the film was estimated to be 1.48 g/cm^3^. The sample has a dark orange color with surface roughness and presents micropores in a range between 30 and 100 μm in diameter, as shown in the micrographs depicted in Figure 1a–c. After dissolution in distilled water, the sample was characterized in terms of its acidity, whereby the measurements gave a pH of 4, meaning that the dissolved film was prominently acidic. The wetting ability of the sample is very high, and the wetting angle is not measurable because it absorbs water immediately, showing a very strong hydrophilic behavior. Our investigations demonstrated that a water drop of 1 μL is absorbed by the surface at a time of about 1 s.

**Figure 2:**
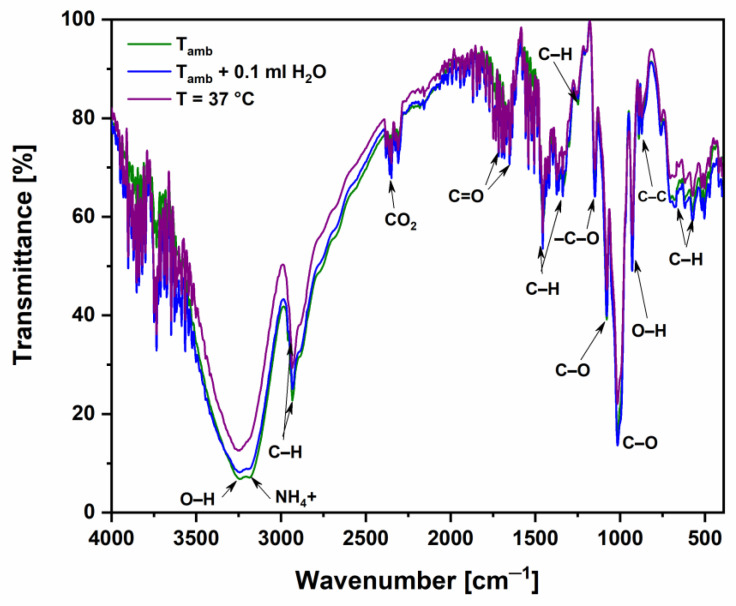
FTIR spectra of the analyzed ODFs (transmittance vs. wavenumber) at different experimental conditions.

The chemical structure of the sample, based on vitamin D3, was investigated using FTIR, monitoring any possible change under different environmental conditions. Figure 2 shows a comparison of the FTIR spectra of the sample relative to its transmittance in the wavenumber region 400–4000 cm^−1^ in dry modality at room temperature (19 °C), at body temperature (pre-heating the sample until it reaches a temperature of 37 °C), and in wetted modality, after absorption of a drop of water (0.1 mL) at ambient temperature. These spectra identify the most important components of the sample as a distinct fingerprint of the analyzed compound. Table 1 shows the identified peaks of functional groups belonging to the various substances present in the analyzed ODFs.

For instance, at 869 cm^−1^, it is possible to recognize the absorption band of the C–C group, while at 1023 cm^−1^, the band is related to the C–O group, both characteristic of vitamin D3 [7]. The absorption peak at 1080 cm^−1^ belongs to the presence of carbohydrate C–O vibration of sugar and organic acids [8]. Peaks at 672 cm^−1^ and at 1464 cm^−1^ are related to C–H bonds, typically found in phospholipids [9]. The peak at 2350 cm^−1^ is related to CO_2_ included in the vitamin D3 film after its exposure to air for some time, as confirmed by our previous work [10]. Finally, peaks at 3180 cm^−1^ and at 3257 cm^−1^ belong to NH_4_^+^ ions and to O–H stretching, respectively [11]. D-mannitol presents peaks associated with the presence of C=O double bonds (i.e., carbonyl groups) between 1730 cm^−1^ and 1645 cm^−1^, as confirmed by the literature [12]. The decrease in these peaks’ transmittance values can be attributed to the absorption of mannitol, which increases with temperature. The peak at 1322 cm^−1^, according to the literature [13], is related to the CH bending (wagging) of ascorbic acid. O–H bending was observed at 910 cm^−1^, probably belonging to the presence of glycerol [14]. The stretching of the –C–O group, corresponding to the band at 1150 cm^−1^, could be associated with encapsulated oil in maltodextrins [15]. Finally, peaks observed at 1239 cm^−1^ and at 564 cm^−1^ are related to the C–O stretching of alcohol and the (FeO) C–H deformation, respectively [16,17]. Comparing the FTIR spectra of the pristine ODF with those acquired under different experimental conditions, it is possible to observe that for most of the characteristic groups identified in the spectra, the transmission drops when heated at 37 °C. This last result suggests that the increase in temperature favors the rapid dissolution of the vitamin film in the oral cavity.

**Table 1:** Functional groups of the complex vitamin D3 ODF identified through FTIR.

Wavenumber [cm^−1^]	Functional Groups	Substance	Ref.
564	C–H deformation	FeO	[16]
869	C–C group	Vitamin D3	[7]
910	O–H bending	Glycerol	[14]
1023	C–O group	Vitamin D3	[7]
1080	C–O vibration	Sugar and organic acids	[8]
1150	–C–O group stretching	Oil in maltodextrines	[15]
1239	C–O stretching	Alcohol	[16]
1322	CH bending (wagging)	Ascorbic acid	[13]
1464	C–H bonds	Phospholipid	[9]
1645–1730	C=O groups (carbonyl)	D-mannitol	[12]
2350	CO_2_		[10]
3180	NH_4_^+^ ion		[11]
3257	O–H stretching		[11]

At the temperature of 37 °C (human body), the transmittance shows a significant reduction, approximately 50%, for the O–H groups around 3250 cm^−1^. This reduction indicates that vitamin D3 is strongly temperature-dependent, which acts as an important parameter for its solubility in aqueous media. This result agrees with the literature data, reporting that stability is strongly dependent on temperature and the type of liquid medium where it is embedded [4]. Higher temperatures, around 37–40 °C, lead to a significant increase in the rate of vitamin D3 degradation in different aqueous media (distilled water, tap water, and ultrapure water or Milli-Q water, Burlington, MA, USA).

**Figure 3:**
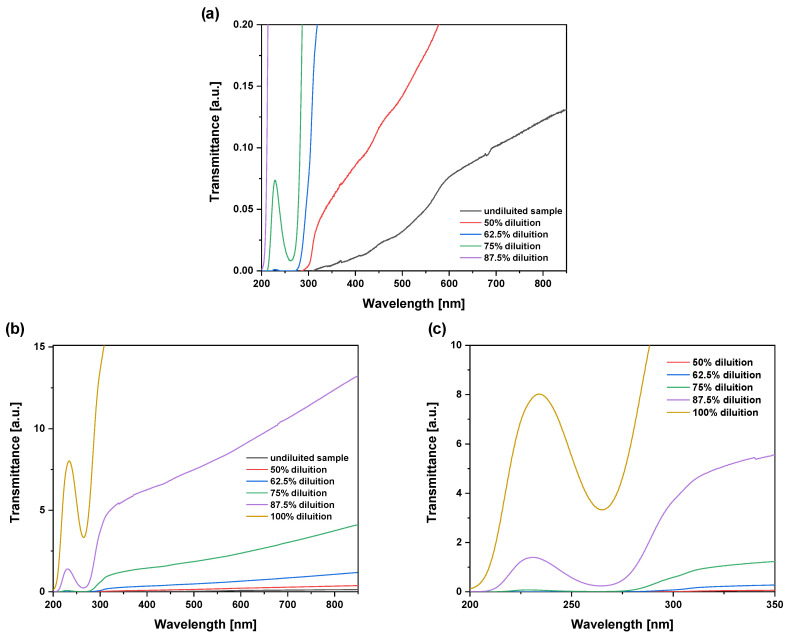
(**a**) Transmittance spectra of the vitamin D3 ODF sample in the wavelength range from 200 to 850 nm before and after its dilution. (**b**) By increasing the transmittance axis values, it is possible to observe the presence of some characteristic transmittance peaks at high dilution values, which are more evident in (**c**), where the spectra are represented in the UV region between 200 and 350 nm.

The ODF sample was also characterized through UV–Vis–NIR spectroscopy in transmittance and absorbance modes. Figure 3a–c shows the transmittance spectra vs. wavelength for different dilutions of the sample. The spectrum in Figure 3a allows us to observe the trend of the undiluted sample and the same ODF after different dilutions in distilled water. By increasing the transmittance axis values of such curves (Figure 3b), it is possible to observe the presence of some characteristic transmittance peaks at high dilution values, which are more evident in the zoomed-in view of Figure 3c, where the spectra are represented in the UV region between 200 and 350 nm.

**Figure 4:**
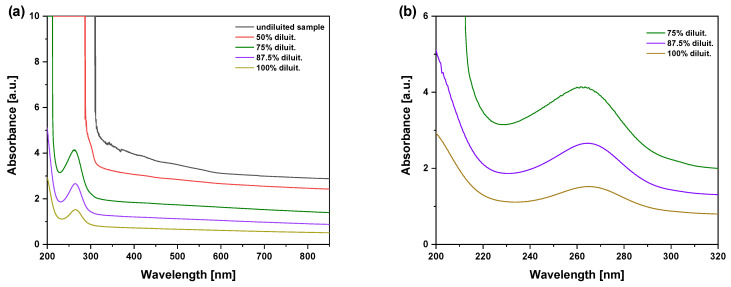
Absorbance spectra vs. wavelength of the ODF sample at different water dilutions (**a**) in a wide wavelength range (from 200 to 850 nm) and (**b**) in the UV region (from 200 to 320 nm).

The spectra indicated that transmittance increases significantly with sample dilution in water. For example, at 500 nm, the transmittance of the pristine sample is only 0.025% (opaque sample), and it increases to 8% at a dilution of 87.5%. In the UV region, within 200–350 nm, the transmittance shows discontinuities and oscillations (see Figure 3c) as a typical trend for the presence of small nanoparticles suspended in water. In order to have more details on these resonant absorption effects due to the presence of nanoparticles in suspension, further measurements of absorbance were performed using the same instrumentation (Figure 4a).

Oscillations are present in the UV region 200–320 nm, around 264 nm, as shown in Figure 4b, indicating resonant absorption for the presence of nanometric particles diluted in the solution. In fact, UV–visible spectroscopy employs light in the visible or ultraviolet range to excite electrons within the sample. When light of a specific wavelength is absorbed by a molecule or nanoparticle, it promotes an electron from a lower-energy orbital to a higher-energy one. The resulting absorbance spectrum provides information about the electronic transitions in the sample. In particular, the absorbance peak at 264 nm wavelength indicates the production of previtamin D3 [18]. Surface plasmon resonance (SPR) sensing consists of monitoring changes in the color scattered off a surface. Surface plasmons are electron oscillations along the interface of a metal and dielectric surface. The frequency of these oscillations is very sensitive to the refractive index of the environment. Thus, in a sensor configuration, the resonance frequency of surface plasmons changes depending on the refractive index of the sample medium. Oscillations in the UV region are typical of nanoparticles with sizes generally below 10 nm [19,20]. The resonant absorption effect observed in the UV region between 200 and 300 nm wavelengths indicates that UV irradiation may be absorbed by vitamin D3 with a possible increment in its degradation due to effects of ionization, radical formation, chemical bond breaking, and enhancement of vitamin solubility.

**Figure 5:**
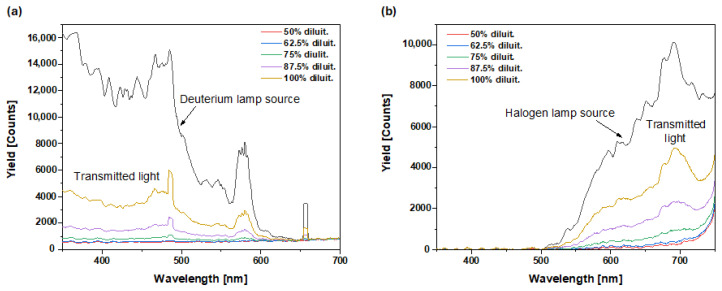
Relative Transmittance vs. wavelength of the ODF sample at different dilutions in water employing (**a**) a deuterium lamp and (**b**) a halogen lamp.

The transmittance in the UV–Vis range was also recorded using the Avantes instrumentation, illuminating the cuvette solution using an optical fiber connected to a deuterium lamp and measuring the transmission with another aligned optical fiber at different sample dilutions in water, as shown in Figure 5a. Similarly, the transmittance in the Vis–NIR range was recorded using the same instrumentation, illuminating the cuvette solution using an optical fiber connected to a halogen lamp and measuring the transmission at different sample dilutions in water, as shown in Figure 5b. The spectra indicate that transmission increases with sample dilution, as expected, and that the transmission is very low in the UV region and at low wavelengths in the visible region. In the IR region and at high visible wavelengths, transmission is slightly higher and increases with the level of dilution. For example, from Figure 5a, at 400 nm wavelength, the transmission *T* is

*T*(400 nm) = *I_T_*/*I*_0_ = 3800/13,500 = 28.1%. (1)

where *I_T_* is the transmitted light and *I_0_* is the incident light. Instead, at 700 nm wavelength, from Figure 5b, the transmission is

*T*(700 nm) = *I_T_*/*I_0_* = 4800/10,000 = 48%. (2)

From such measurements, it is possible to evaluate the absorption coefficient *μ* (cm^−1^) of a 1 cm-thick cuvette (Δ*x*) solution for 100% dilution in the visible wavelength range. At the two above -mentioned wavelengths, the following values are obtained:

(3) $$\mu \left(400\;\mathrm{nm}\right)=\frac{1}{\Delta x}\;\ln\frac{I_{0}}{I_{T}}=\frac{1}{1\mathrm{cm}}\ln\frac{1}{0.281}=1.27\;{\mathrm{cm}}^{-1}$$

and

(4) $$\mu \left(700\;\mathrm{nm}\right)=\frac{1}{\Delta x}\;\ln\frac{I_{0}}{I_{T}}=\frac{1}{1\mathrm{cm}}\ln\frac{1}{0.4}=0.73\;{\mathrm{cm}}^{-1}.$$

**Figure 6:**
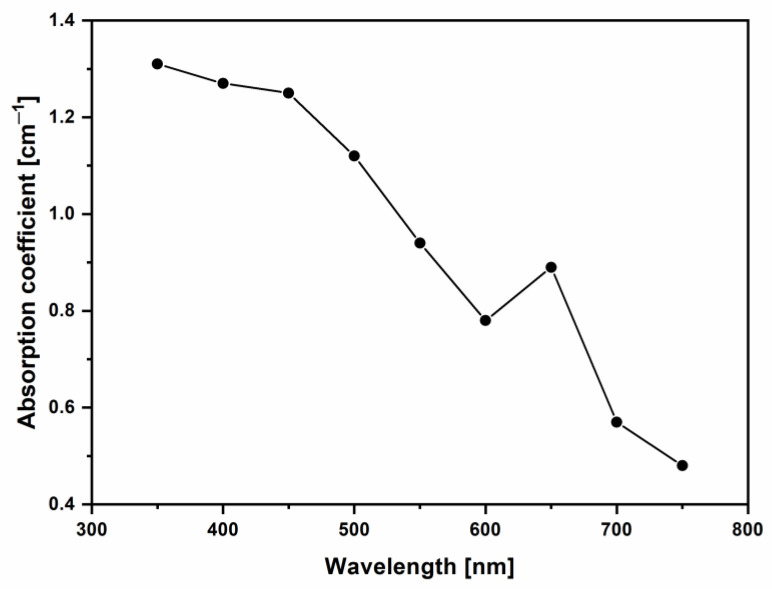
Absorption coefficient of vitamin D3 ODF sample in water (100% dilution) vs. wavelength.

By performing this calculation for different wavelength values, it is possible to estimate the absorption coefficient vs. wavelength by obtaining the results reported in Figure 6.

Thus, the absorption coefficient decreases from the visible range into the near IR region, according to the absorbance data reported in Figure 4. However, this calculation can be performed for all analyzed wavelengths in the range between UV, at about 300 nm, and NIR, at about 800 nm, by evaluating the absorption coefficients using the Lambert–Beer law.

## 3. Materials and Methods

### 3.1. Investigated Sample (Vitamin D3 ODFs)

**Figure 7:**
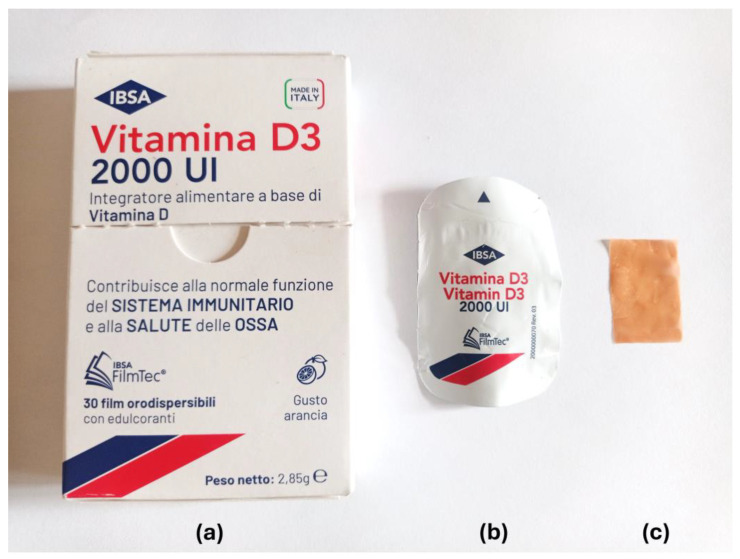
Primary (**a**) and secondary (**b**) packaging of Vitamin D3 orodispersible films and a film (**c**).

Changes in the chemical structure (bond scission/forming) of the ODF copolymer after UV irradiation were monitored using ATR-FTIR spectroscopy. A picture of the films and their commercial package is shown in Figure 7, while their main composition is reported in Table 2.

Moreover, the ODFs contain traces of other substances, such as maltodextrins, glycerol, water, mannitol, extra virgin olive oil, Orange flavor, Polyvinylpyrrolidone vinyl acetate copolymer, ascorbic acid, Alphatocopherol, Polyoxyethylene sorbitan monooleate, Titanium dioxide, Iron oxides and hydroxides, and Sucralose. The stability study showed that vitamin D3 was ≥90% after 3 months at 40 °C. The film disintegrated in less than 1 min, and the vitamin D3 released was ≥75% after 15 min [3]. We examined 10 ODF samples, and we reported the average values obtained.

### 3.2. Lamp Sources

Measurements of absorbance and transmittance in the UV–Vis–IR range were obtained from a dissolved solution in a cuvette by measuring the optical properties under the Avantes deuterium and halogen lamps AvaLight-DH-S (Apeldoorn, the Netherlands) equipped with an integrated TTL shutter. The deuterium lamp emits in the wavelength regions of 200–700 nm with high emission in the UV region, while the halogen lamp covers the region of 500–800 nm with maximum yield in the IR region. The light is transported to the solution through a quartz optical fiber having a 20-micron diameter. The irradiations were performed in air at room temperature (20 °C), 1 atm pressure, and at a humidity rate of 50%. The samples were dissolved in a cuvette containing 3 cc of distilled water and placed at about 3 cm distance from the lamp.

### 3.3. Optical Spectroscopy

The effects of ultraviolet radiation on the sample were investigated using an FTIR spectrometer, Jasco 4600 (JEOL, Akishima, Japan), with attenuated total reflectance (ATR) coupled with Fourier transform spectroscopy to investigate chemical changes in the sample before and after UV irradiation at different exposure times. The ATR-FTIR spectra were collected in the range from 4000 to 390 cm^−1^ under normal atmospheric conditions, without vacuum. The optical properties were also investigated using a double-beam UV–Vis–NIR spectrophotometer (Jasco Mod. V-750, Jasco-Europe, Lecco, Italy) in the wavelength range of 300–900 nm. The spectra were acquired with atmospheric correction.

Finally, optical measurements in reflectance mode were performed using an AvaSpec-2048 spectrophotometer (Avantes) with UB-600 lines/mm grating and a bandwidth of 195–757 nm. The source designed by Avantes was a deuterium halogen lamp AvaLight-DH-S, with an integrated TTL shutter. Optical microscopy (Paralux, Geispolsheim, France) was employed for morphology investigation. All UV irradiations were performed under the same experimental conditions (3 cm distance lamp—IOL maintained constant, room temperature (22 °C), 1 atm pressure, and 45% humidity rate).

## 4. Conclusions

In this work, we employed simple and low-cost UV–Vis–IR spectroscopic techniques to study and characterize vitamin D3. This approach serves as a digital fingerprint for the biological material that contains vitamin D3 and can be used to search for it in food, drugs, and environmental samples. The present work employed spectroscopic techniques (FTIR and UV–Vis) to investigate the structure of commercial vitamin D3 orodispersible films (ODFs) and their intermolecular interaction with other excipients, evidencing their optical properties. The study of hydrogen bonding, molecular rearrangements, and crystalline transformations will guide the selection of appropriate excipients and processing techniques.

These films are developed using various excipients and techniques to ensure stability and bioavailability. For instance, using maltodextrin as a primary film-forming ingredient has shown promising results in producing stable, effective ODFs for vitamin D3 supplementation. The innovative nature of ODFs allows them to be used in both therapeutic and supplemental contexts, providing a versatile solution for enhancing vitamin D3 intake. We demonstrated that this, in turn, ensures the stability and bioavailability of vitamin D3. Furthermore, UV–Vis analysis of photostability will provide valuable insights into potential degradation pathways under simulated environmental conditions. Moreover, ODFs offer an innovative delivery method that dissolves rapidly in the mouth, requiring only a small amount of saliva. This makes them particularly beneficial for populations with swallowing difficulties, such as the elderly and children. Recent studies have highlighted the superior dosing accuracy, rapid onset of action, and improved patient compliance associated with ODFs compared to conventional dosage forms.

Integrating insights from physics and biology to enhance the clinical application of vitamin D absorption through ODFs can lead to the development of precise self-administration guidelines. Indeed, to employ ODFs effectively, they should be placed centrally on the tongue. The user should then produce saliva and keep their mouth closed without swallowing for at least five minutes. For patients who can drink water before the procedure, this can help dissolve the product more rapidly.

Although the present study characterized vitamin D3 only in water solutions, the employed study could be applicable to the accurate estimation of routine analysis of vitamin D in food, feed, pharmaceuticals, and environmental samples in the drug industry and other recognition fields.

Work is in progress in order to present vitamin D3 degradation quantification as a function of absorbed UV exposure dose. This study will play a crucial role in validating the quality and confirming the solubility of ODFs, ultimately enhancing their formulation, production efficiency, and the reliable delivery of effective therapeutic doses.

## Figures and Tables

**Table 2:** Composition of the investigated vitamin D3 ODF.

Average Values for 1 ODF	
Fats	3.8 mg
-of which saturated fats	0.5 mg
Carbohydrates	79.3 mg
-of which sugars	0.9 mg
Proteins	0.1 mg
Salt	<0.1 mg
Vitamin D3	50 μg

## Data Availability

The experimental data used to support the findings of this study are included in this article.
